# A biomechanical comparison of closed-reduction osteosynthesis techniques for fractures of the ulnar styloid

**DOI:** 10.1186/s13018-026-07188-2

**Published:** 2026-08-28

**Authors:** Jonas Eck, Inska Onken, Julian P. Maier, Kilian Reising, Nikos Karvouniaris, Jörg Bayer, Hagen Schmal, Ferdinand C. Wagner

**Affiliations:** 1 Department of Orthopedics and Trauma Surgery, University Medical Center Freiburg, Freiburg, Germany; 2 G.E.R.N. Tissue Replacement, Regeneration & Neogenesis, Department of Orthopedics and Trauma Surgery, University Medical Center Freiburg, Freiburg, Germany; 3 Faculty of Medicine, University of Freiburg, Freiburg, Germany; 4 Department of Trauma and Orthopaedic Surgery, Schwarzwald-Baar Hospital, Villingen-Schwenningen, Germany; 5 Department of Orthopedic Surgery, University Hospital Odense, Odense, Denmark

**Keywords:** Ulnar styloid, Wrist, Trauma surgery, Hand surgery, Fracture, Biomechanics, Headless compression screw

## Abstract

**Background:**

Ulnar styloid fractures (USF) are frequently underestimated and can contribute to clinically relevant wrist instability and impaired functional outcomes. Several osteosynthesis techniques are available for the treatment of unstable USF. This study aims to compare the biomechanical stability of fixation methods applicable to closed reduction — specifically K-wire and headless compression screw fixation — to support clinical decision-making.

**Methods:**

This biomechanical in vitro study was conducted using 30 specimens of artificial ulnae. USF were simulated through a standardized osteotomy at the base. Three groups of ten specimens each were created to compare three methods of osteosynthesis: One 1.4 mm (mm) Kirschner-wire (K-wire) (Group 1KW), two 1.0 K-wires (Group 2KW) or one 2.0 cannulated headless screw (Group CHS). Each construct underwent 1,000 load cycles up to 50 newtons (N) followed by load-to-failure testing, which was defined as displacement ≥ 2 mm. Displacement [mm], stiffness [N/mm] and load to failure [N] were recorded and compared among the groups.

**Results:**

The mean fracture displacement after 1,000 cycles was 0.58 mm (SD 0.37) in the 1KW group, 0.23 mm (SD 0.09) in the 2KW group and 0.09 mm (SD 0.03) in the CHS group. Significant group differences could be shown between CHS and 1KW (*p* < 0.01) as well as between CHS and 2KW (*p* = 0.017). The mean construct stiffness was 269.95 (SD 45.72) N/mm in the 1KW group, 484.08 (SD 207.60) N/mm in the 2KW group and 730.95 (SD 241.42) N/mm in the CHS group. Significant group differences could be shown between CHS and 1KW (*p* < 0.01). The mean load-to-failure was 136.82 (SD 68.86) N in the 1KW group, 181.60 (SD 28.09) N in the 2KW group and 204.17 (SD 63.10) N in the CHS group. Significant group differences could be shown between CHS and 1KW (*p* = 0.013).

**Conclusion:**

Ulnar styloid refixation with a headless compression screw provides superior axial stability compared to single or double K-wire fixation. This should be considered in the treatment of unstable USF.

## Background

Ulnar styloid fractures (USF) represent a clinical challenge in everyday practice. The prevalence of concomitant USF in distal radius fractures is reported to be as high as 55–58% [1–3]. Osteosynthesis of these fractures is technically demanding due to the small fragment size, and its clinical benefit remains a matter of debate. Non-union of USF has been correlated with poorer functional outcomes and higher pain scores [2]. In a retrospective study, Kang et al. analysed patients with operatively treated USF using either tension band wiring (TBW) or hook plate fixation and reported a high union rate of 96% [4]. In contrast, conservative treatment of USF resulted in a considerably high non-union rate (26%) as reported by Chen and colleagues in a series of distal radius fractures which were treated by external fixation and Kirschner-wires (K-wires) [3].

**Fig. 1:**
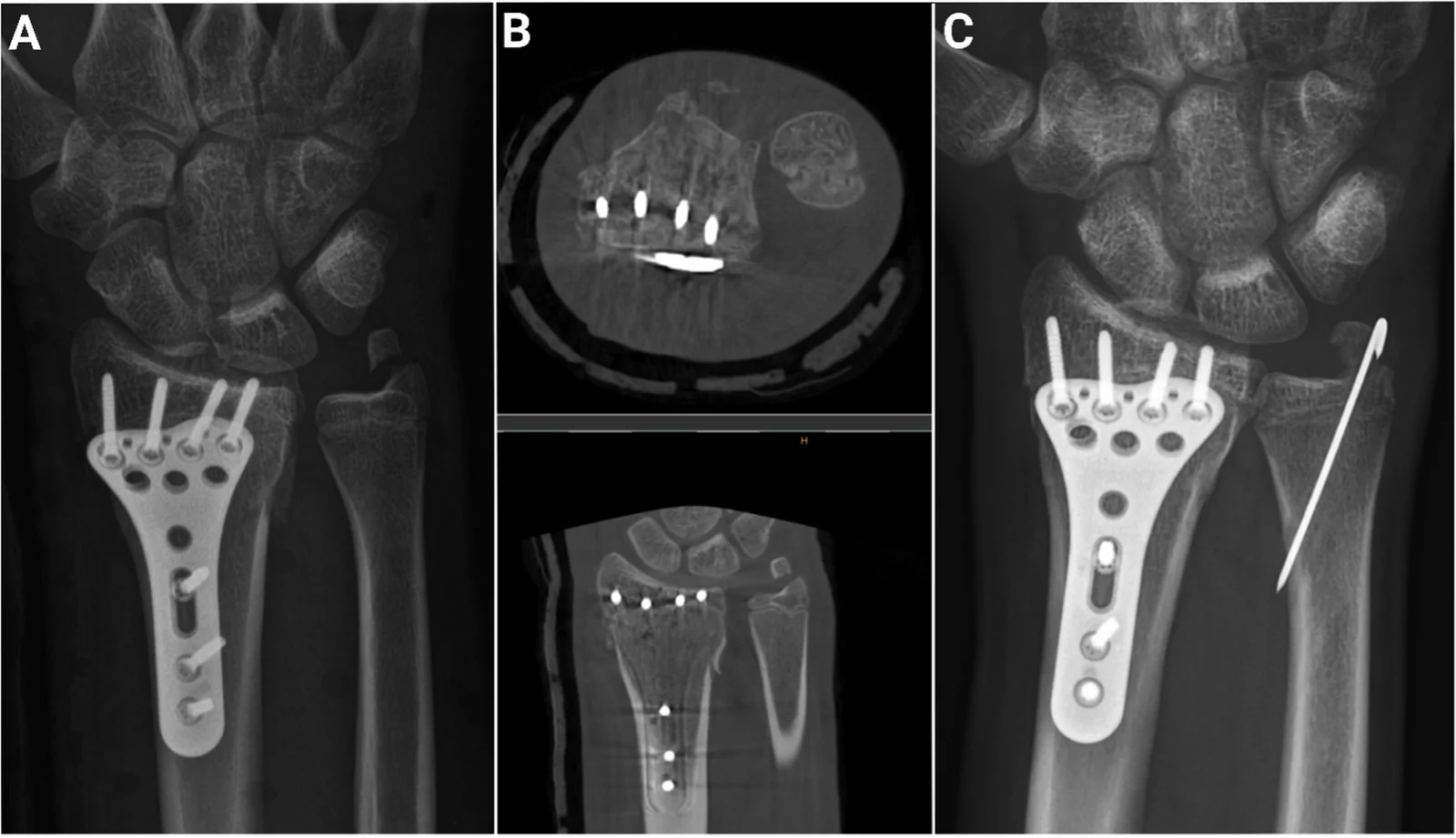
Demonstration of a clinical case. The distal radius fracture was stabilized using plate osteosynthesis (**A**). Postoperative computed tomography (CT) demonstrated persistent instability of the DRUJ (**B**). Restoration of distal radioulnar joint stability and radiological signs of early consolidation at 6 weeks postoperatively were achieved by single K-wire osteosynthesis of the ulnar styloid (Fig. 1**C**)

Previous biomechanical studies have shown that increasing fragment size of the ulnar styloid is associated with instability of the distal radioulnar joint (DRUJ) [5, 6]. Figure 1 illustrates a representative clinical case supporting these biomechanical findings. Although the distal radius fracture was stabilized using plate osteosynthesis (Fig. 1A), postoperative computed tomography (CT) demonstrated persistent incongruence of the DRUJ (Fig. 1B), suggesting instability. Pathological joint instability is generally believed to alter load distribution, thereby increasing the risk of degenerative changes. Thus, from a biomechanical perspective, osteosynthesis of relevant USF appears reasonable to restore physiological load transfer and prevent secondary joint degeneration.

Various fixation techniques — including two K-wires, a compression screw, TBW, and suture anchor fixation — have been evaluated in cadaveric studies [7]. However, in clinical practice, concomitant USF are often treated with a single K-wire, primarily due to the small fragment size, the possibility of closed reduction and technical simplicity of this method. This technique was applied in the presented case, resulting in restoration of distal radioulnar joint stability and radiological signs of early consolidation at 6 weeks postoperatively (Fig. 1C). This approach, however, has not yet been biomechanically evaluated.

The aim of this study is to assess the biomechanical performance of different osteosynthesis techniques which allow closed reduction for the treatment of USF. The findings of this investigation may contribute to more evidence-based clinical decision-making in the management of these underestimated injuries.

## Methods

**Fig. 2:**
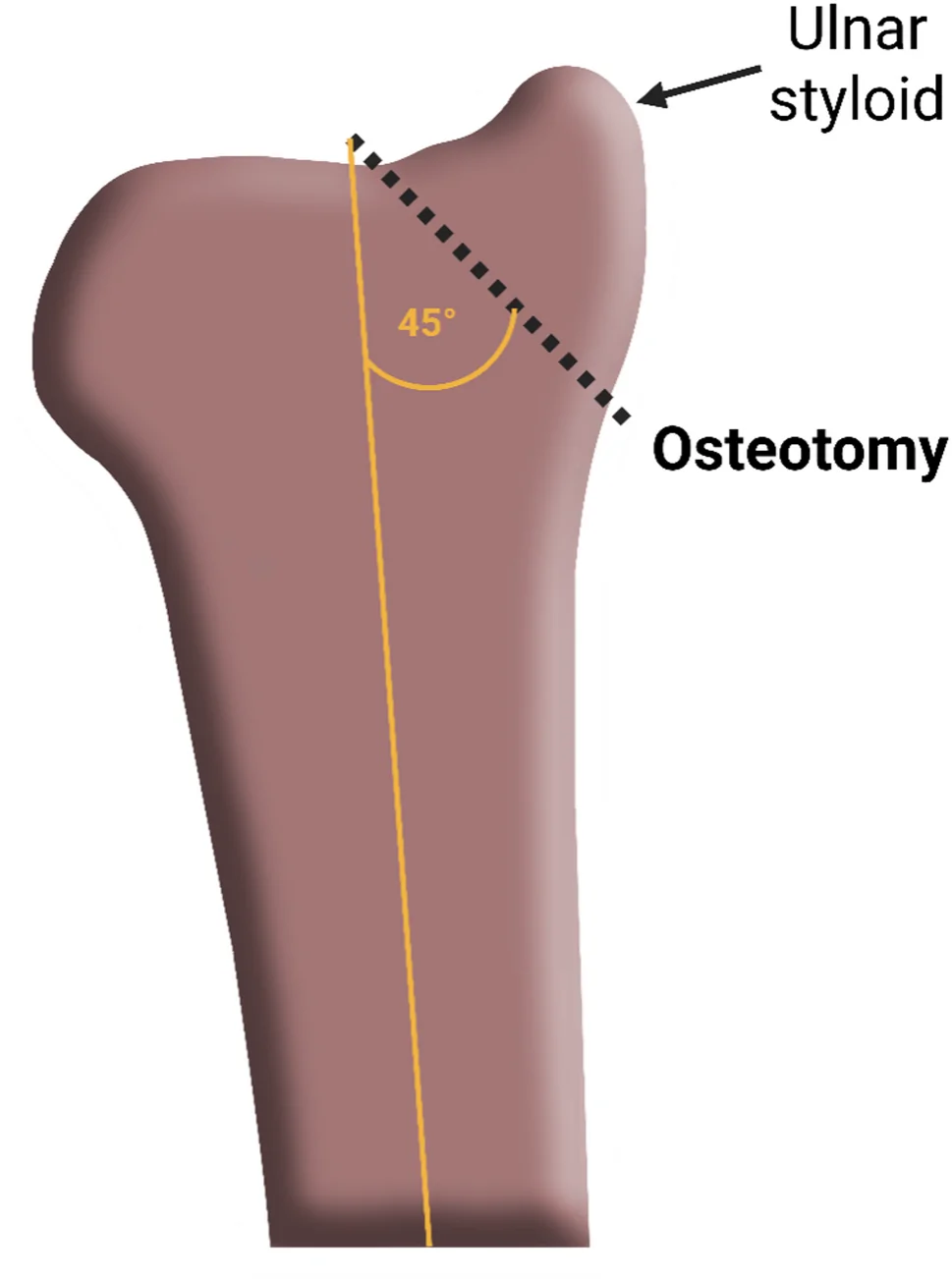
Illustration of distal ulna and the localisation of the performed osteotomy, imitating an ulnar styloid fracture

Thirty biomechanically validated synthetic ulnae (Ulna, *absolute™ 4th Gen.*, 17 PCF Solid Foam Core, Large, Sawbones^®^, Vashon, Washington, USA) were used in this biomechanical in vitro study. A standardized osteotomy with a 45° angle relative to the shaft including the fovea was performed to simulate an AO type 2U3A1.2 ulnar styloid fracture (USF) using a diamond coated band saw with a saw blade thickness of 0.2 millimetres (mm) (Fig. 2). This fracture configuration has previously been shown to result in significant instability of the distal radioulnar joint (DRUJ) [5, 6]. The specimens were randomly allocated into three groups (*n* = 10 each), representing different osteosynthesis techniques:

- Group 1 (1KW): fixation with a single K-wire (Ø 1.4 mm; DePuy Synthes, Johnson & Johnson).
- Group 2 (2KW): fixation with two K-wires (Ø 1.0 mm each; DePuy Synthes, Johnson & Johnson).
- Group 3 (CHS): fixation with a cannulated headless compression screw (CCHS, Ø 2.0 mm; DePuy Synthes, Johnson & Johnson) placed over a guide wire according to the manufacturer’s instructions.

The single 1.4 mm K-wire configuration (Group 1) was selected to reflect the most commonly applied technique for closed reduction of ulnar styloid fractures in our clinical practice, as described in the Background. The two-K-wire configuration (Group 2) using 1.0 mm wires was chosen to approximate the setup described by Maniglio et al. 2021 [7].

**Fig. 3:**
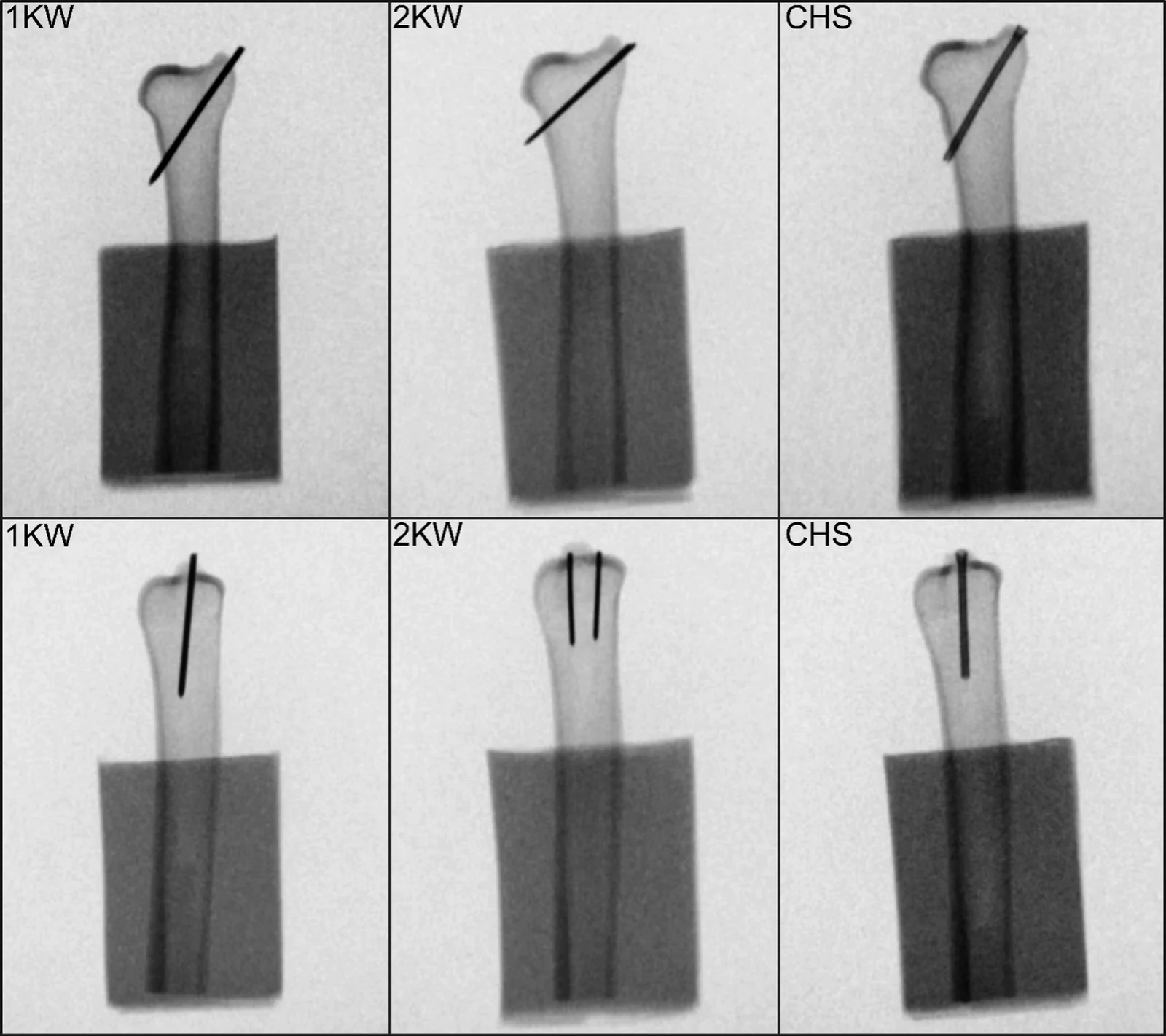
X-Rays of Sawbones after osteosynthesis of the performed osteotomy in ap-view (top row) and lateral-view (bottom row). 1KW = 1 K-Wire, 2KW = 2 K-Wires. CHS = Cannulated compression screw

**Fig. 4:**
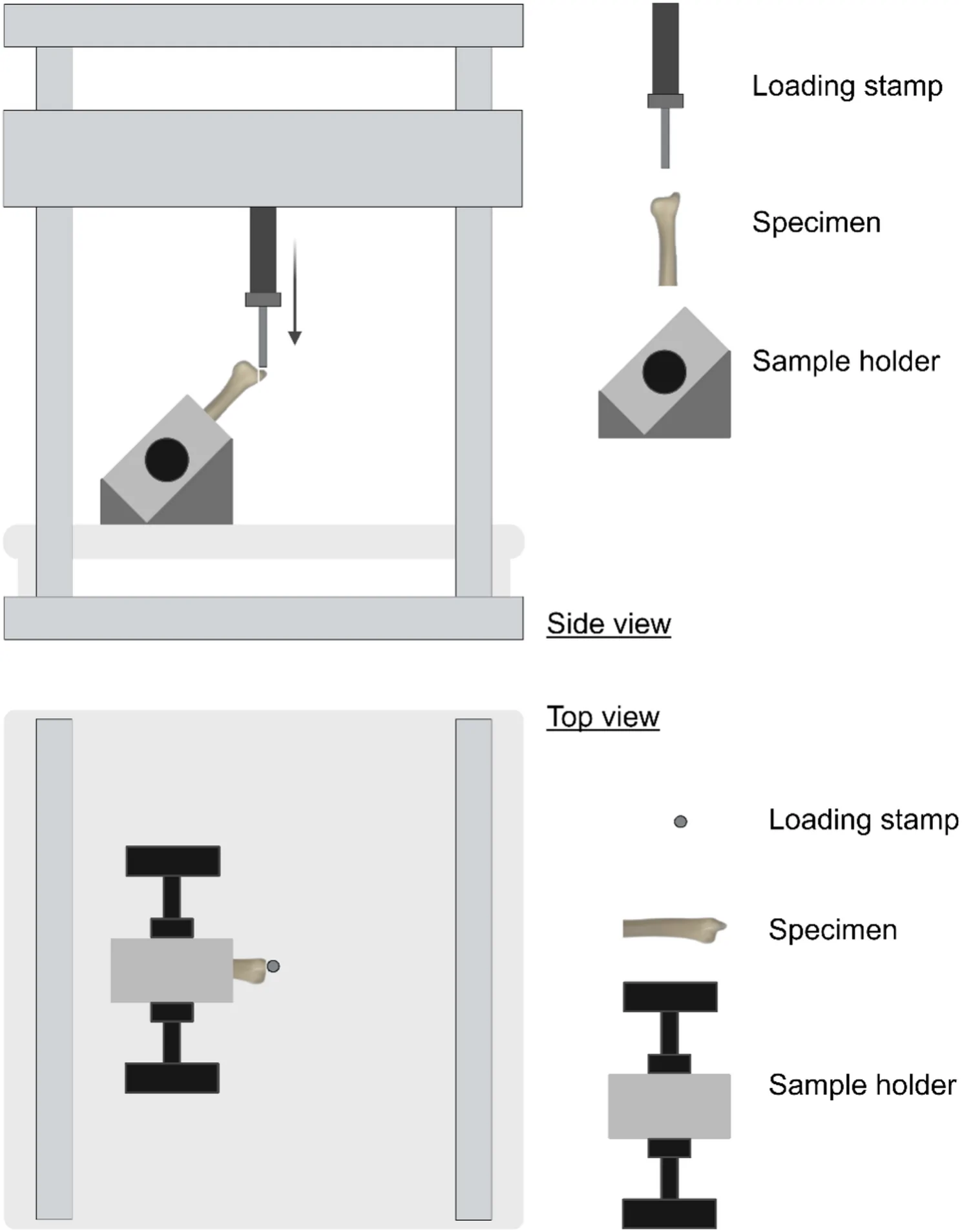
Schematic illustration of testing setup. The setup is shown in a side and top view. Created in BioRender (https://app.biorender.com/)

In all groups, fixation included both cortices, and adequate reduction and implant placement were verified radiographically (Fig. 3). The distal ends of all specimens were trimmed to a uniform length of 8.5 cm and embedded 5 cm deep in polymethylmethacrylate (PMMA; Technovit 3040, Heraeus Kulzer GmbH, Wehrheim, Germany) to ensure secure and standardized mounting in the testing apparatus, as previously described (Fig. 4) [8]. Biomechanical testing was performed using a servo-hydraulic testing machine (Amsler HC10, Zwick/Roell, Ulm, Germany) equipped with a cylindrical loading stamp (10 mm diameter). The load was applied parallel to the fracture plane, as illustrated in Fig. 3. All specimens first underwent cyclic loading of 1,000 cycles at a frequency of 0.1 Hz with a sinusoidal force ranging from 5 to 50 N. The optimal load was determined in preliminary test trials. Fracture displacement was quantified by measuring the change in stamp position throughout the experiment. The maximum fracture displacement after 1,000 loading cycles was recorded and reported in millimetres. Construct stiffness (N/mm) was determined as mean stiffness of the initial 50 loading cycles. Subsequently, each specimen underwent load-to-failure testing, defined as the force (N) at which 2 mm of fracture displacement occurred. This procedure was performed under a continuously increasing load at a rate of 2 N/s.

### Statistical analysis

Statistical analysis was performed using Microsoft Excel (Version 2508, Build 19127.20264, 01 November 2025) and an open Python environment by Jupyter^®^. Continuous data are reported as arithmetic means with standard deviations (SD). Categorical variables are presented as absolute frequencies. Relative frequencies are reported as percentages. Shapiro-Wilk test showed no normal distribution. Group differences were analysed for statistical significance with a Kruskal Wallis test and Dunn’s test with Bonferroni adjustment for post-hoc analysis. Statistical significance was assumed at a p-value of < 0.05.

## Results

Of 30 specimens, 28 underwent complete cyclic loading with 1,000 cycles. One specimen of the 1KW group and one of the 2KW group did not complete the 1,000 cycles due to machine-related malfunction resulting in unintended excessive force transmission and premature construct failure. These failures occurred during the cyclic loading phase prior to meaningful data acquisition and were not attributable to implant or fracture-related failure. Both specimens were excluded from all analyses. The resulting group imbalance (*n* = 9 vs. 9 vs. 10) is acknowledged as a potential limitation.

**Fig. 5:**
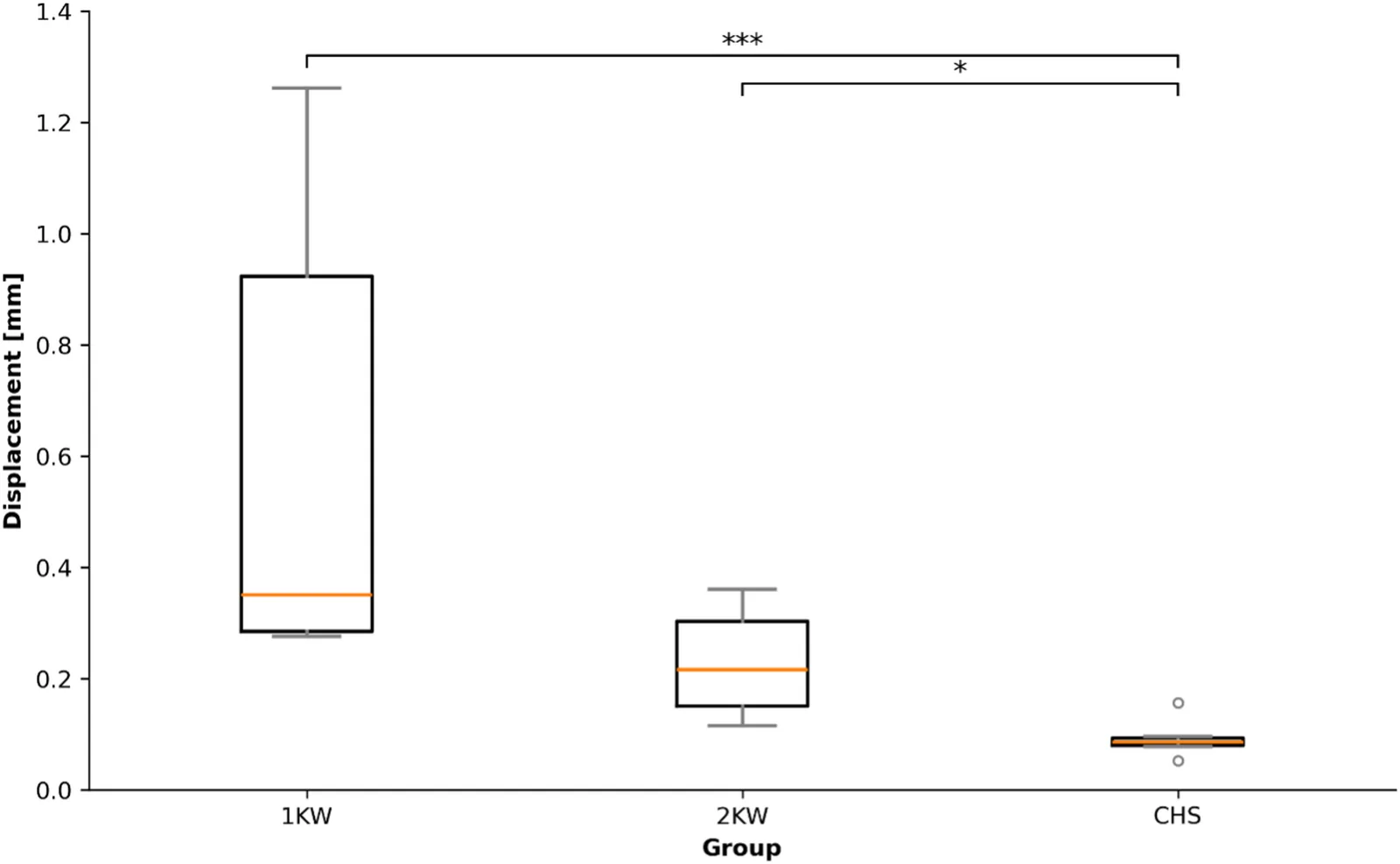
Box plot of the fracture displacement in Millimetres after 1,000 cycles of cyclic loading divided by groups (1KW = 1 K-Wire, 2KW = 2 K-Wires. CHS = Cannulated headless screw). Shown are the median, the interquartile range (1st and 3rd quartiles), and whiskers representing 1.5 times the interquartile range. Outliers are marked as circles. Significant group differences are indicated by * (*p* = 0.017) and *** (*p* = 0.00003)

The mean fracture displacement after 1,000 cycles (Fig. 5) was 0.58 (SD 0.37) mm in the 1KW group, 0.23 (SD 0.09) mm in the 2 KW group and 0.09 (SD 0.03) mm in the CHS group. Significant group differences could be shown between CHS and 1KW (*p* = 0.00003) as well as between CHS and 2KW (*p* = 0.017).

**Fig. 6:**
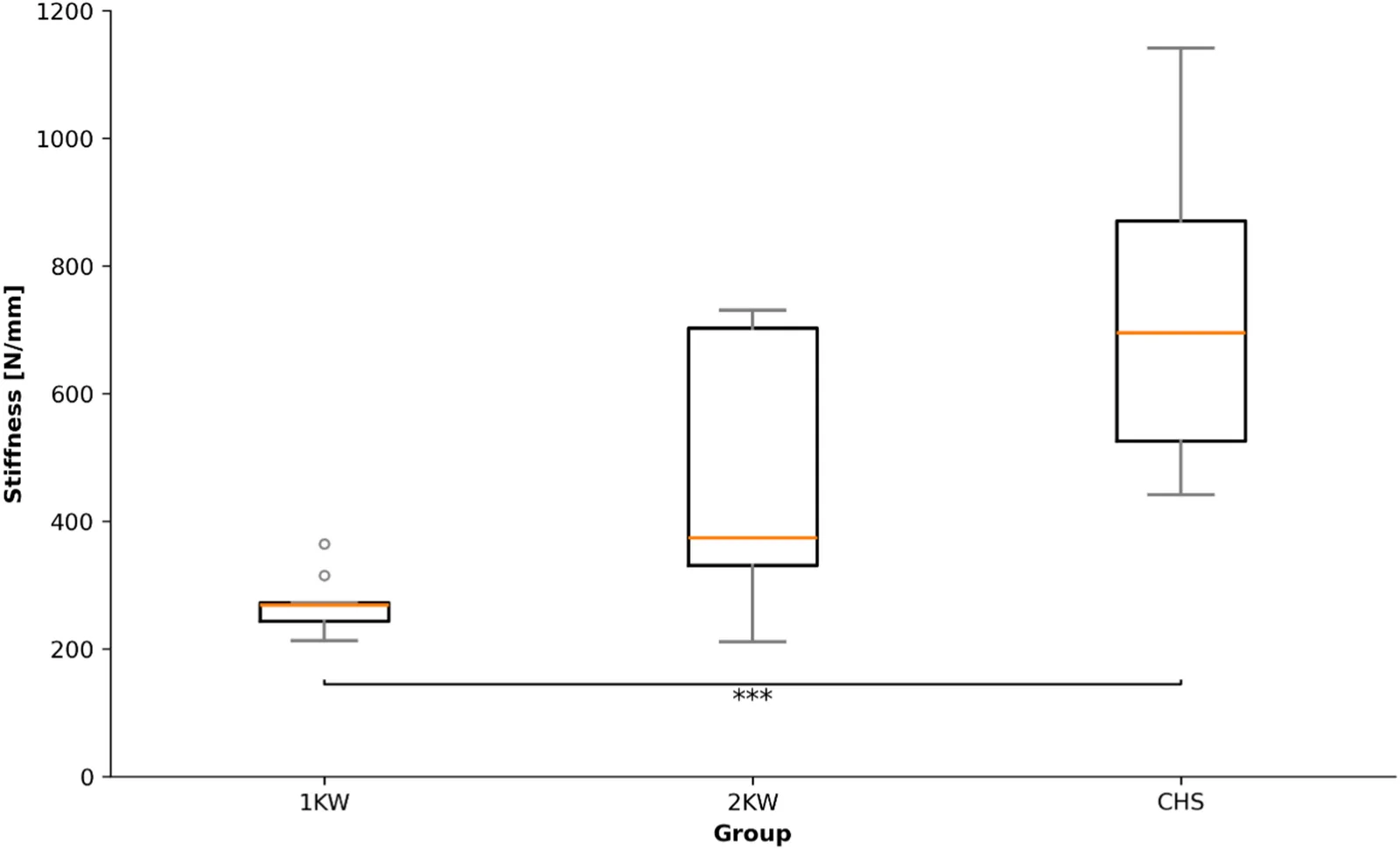
Box plot of the construct stiffness in Newton/Millimetre divided by groups (1KW = 1 K-Wire, 2KW = 2 K-Wires. CHS = Cannulated headless screw). Outliers are marked as circles. Significant group difference is indicated by *** (*p* = 0.0002)

The mean construct stiffness (Fig. 6) was 269.95 (SD 45.72) N/mm in the 1KW group, 484.08 (SD 207.60) N/mm in the 2 KW group and 730.95 (SD 241.42) N/mm in the CHS group. Significant group differences could be shown between CHS and 1KW (*p* = 0.0002).

**Fig. 7:**
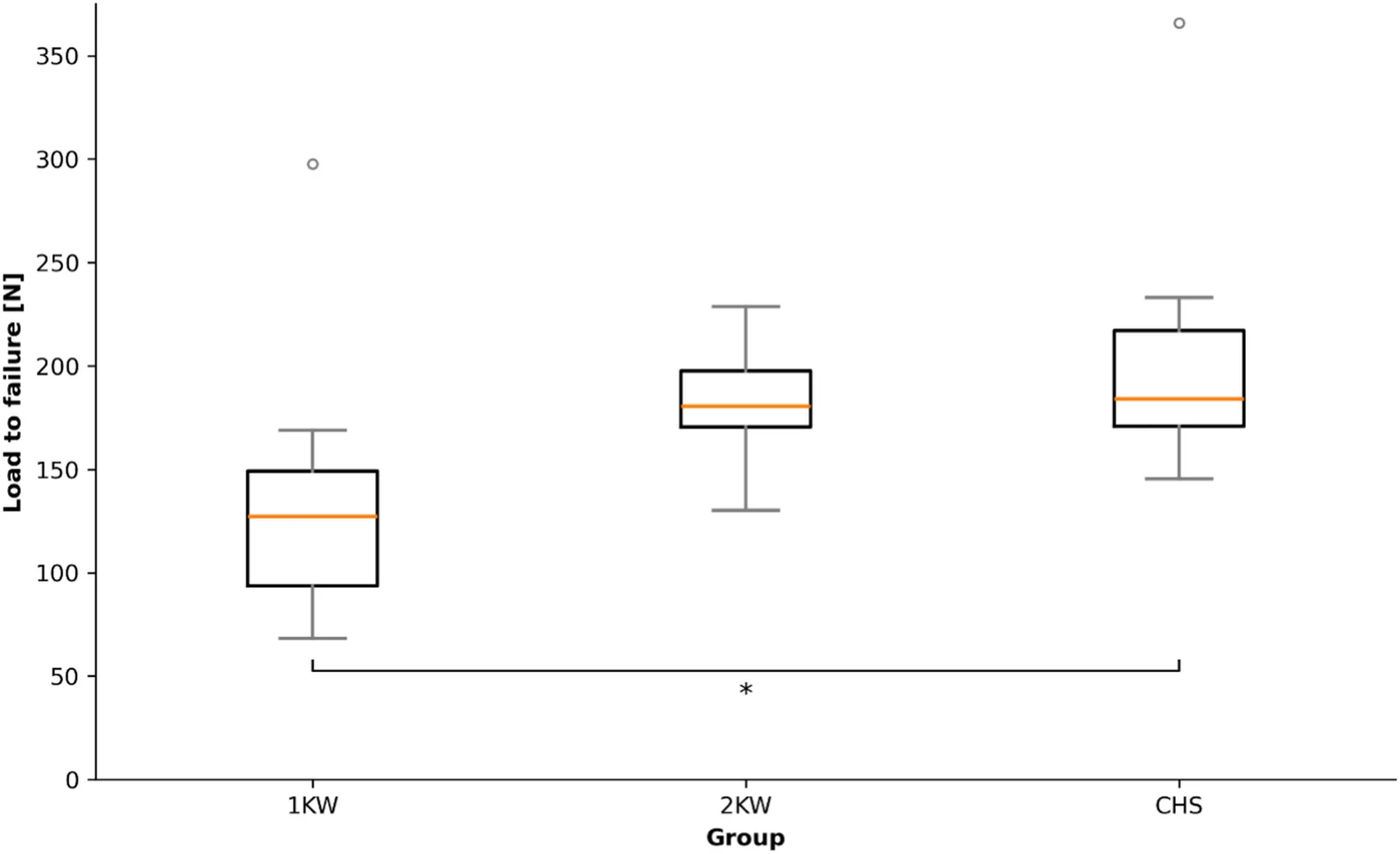
Box plot of the Load to failure in Newton at a defined fracture displacement of 2 Millimetres divided by groups (1KW = 1 K-Wire, 2KW = 2 K-Wires. CHS = Cannulated headless screw). Outliers are marked as circles. Significant group difference is indicated by * (*p* = 0.012)

The mean Load to failure (at a defined displacement of 2 mm) (Fig. 7) was 136.82 N (SD 68.86) in the 1KW group, 181.60 N (SD 28.09) in the 2 KW group and 204.17 N (SD 63.10) in the CHS group. Significant group differences could be shown between CHS and 1KW (*p* = 0.012).

**Table 1:** displays a numeric summary of results

Group comparison	Mean (SD)	*p*-Value
*Displacement*	*[mm]*	
1KW – 2KW	0.58 (0.37) – 0.23 (0.09)	0.32575
1KW – CHS	0.58 (0.37) – 0.09 (0.03)	0.00003***
2 KW – CHS	0.23 (0.09) – 0.09 (0.03)	0.01747*
*Stiffness*	*[N/mm]*	
1KW – 2KW	269.95 (45.72) – 484.08 (207.60)	0.07626
1KW – CHS	269.95 (45.72) – 730.95 (211.07)	0.00020***
2 KW – CHS	484.08 (207.60) – 730.95 (211.07)	0.27119
*Load to Failure*	*[N]*	
1KW – 2KW	136.82 (68.86) – 181.60 (28.09)	0.05219
1KW – CHS	136.82 (68.86) – 204.17 (63.10)	0.01246*
2 KW – CHS	181.60 (28.09) – 204.17 (63.10)	1.0

Table 1: Group comparisons of mean values and standard deviations (SD). 1KW = 1 K-Wire, 2KW = 2 K-Wires. CHS = Cannulated headless screw. Pairwise testing was performed using Dunn’s test with Bonferroni correction.

Construct failure could be divided into material failure and shattering of the ulnar styloid. Interestingly, in both the 1KW and 2KW groups, three specimens each displayed shattered ulnar styloids, whereas CHS group exhibited this failure pattern in only one specimen.

## Discussion

The main findings of this study demonstrate that compression screw fixation provides superior stability compared to single K-wire osteosynthesis in USF, both in terms of displacement under cyclic loading and load to failure. Fixation with two K-wires also exhibited significantly greater displacement after cyclic loading compared to compression screw fixation, although the load to failure did not differ significantly. While dual K-wire fixation tended to provide greater stability than single K-wire fixation, this difference was not statistically significant. Overall, these results indicate that although double K-wire fixation may confer partial mechanical benefits, compression screw fixation offers a more reliable and stable construct for the treatment of USF.

It should be acknowledged that the finding of superior biomechanical performance of compression screw fixation over K-wire constructs is consistent with established biomechanical principles of interfragmentary compression and therefore represents a confirmatory rather than fundamentally novel observation. Nevertheless, the present study adds quantitative biomechanical data, thereby contributing incremental evidence to the existing literature.

Concomitant USF associated with distal radius fractures have been linked to poorer outcomes, including increased pain and reduced functional scores, particularly in patients with operatively treated distal radius fractures [9]. Other studies, however, found no significant differences in functional outcomes between surgical and non-surgical management of USF, though conservatively treated fractures exhibited higher rates of non-union but lower complication rates [10]. Non-union has been shown to be more likely in initially displaced fractures, and ulnar styloid fractures with displacement exceeding 2 mm carry a significantly increased risk of non-union [11]. Despite these findings, no universal consensus exists regarding the indication for surgical fixation of USF.

Nakamura et al. investigated DRUJ instability following various osteotomy configurations in a cadaveric model, including tip, middle, base-horizontal, and base-oblique cuts [6]. They found that base-oblique osteotomies - comparable to those performed in the present study - resulted in significant DRUJ instability [6]. This relationship was also demonstrated in a cadaveric study by Cheema et al., in which stress radiographs revealed increased DRUJ-instability following osteotomy at the base of the ulnar styloid [12].

Beyond potential DRUJ instability, USF may also influence wrist joint stability. Clinically, distal radius fractures associated with ulnar styloid fragments ≥ 7 Millimetres (mm) have been linked to a higher incidence of positive triangular fibrocartilage complex (TFCC) grind tests [13]. Consequently, secondary degenerative changes due to chronic ulnar instability must also be considered.

Several fixation techniques have been described, including tension band wiring (TBW), hook plate fixation, K-wires, and compression screws [4, 7, 10]. TBW and hook plate fixation are technically more demanding than single-screw or K-wire fixation, require larger incisions, and may cause greater soft-tissue irritation and implant associated complications due to their larger implant profile [14]. In contrast, K-wires are inexpensive, widely available, and technically simple to use, making them an attractive option in daily clinical practice.

However, the present study demonstrates that single K-wire fixation is biomechanically inferior in both cyclic loading and load-to-failure testing. Among the tested constructs, compression screw fixation proved to be the stiffest and most resistant to displacement and construct failure. Even in this simplified setup without rotational forces, a single K-wire failed to achieve stability comparable to that of a compression screw. Although the use of two K-wires may partially compensate for rotational instability, this configuration still exhibited inferior axial cyclic stability compared to screw fixation. Moreover, the higher rate of shattered ulnar styloids in the 1KW and 2KW groups, even under lower forces at the load to failure, suggests that K-wire fixation may contribute to weakening of the bone compared to headless compression screw fixation.

In their comparative cadaveric study, Maniglio et al. found that two K-wires and TBW provided superior resistance to rotational instability relative to compression screws and suture anchors [7]. Their testing protocol employed cadaveric specimens and allowed separate application of rotational and translational forces at the wrist. Most soft tissues were removed, and the osteotomy was performed at the base of the ulnar styloid, including the fovea, creating a fracture pattern comparable to that described in our study. This type of basal fracture has been shown to produce marked instability of the distal radioulnar joint (DRUJ) in a previous study by the same authors [5]. Their fixation used one 0.8 mm and one 0.9 mm K-wire, whereas our study employed two 1.0 mm K-wires. Furthermore, in their experimental design, the four fixation techniques (K-wires, TBW, compression screw, and suture anchor) were tested sequentially on the same specimens in identical order. This approach raises the possibility that cumulative weakening of the overall construct may have influenced the results of the latter techniques, potentially underestimating their stability. That possible limitation was avoided in our protocol by using a different specimen for each osteosynthesis.

The experimental setup does not account for biological fracture healing or the complex anatomical and soft-tissue structures of the wrist. Specifically, rotational forces occurring during forearm pronation-supination and TFCC-related traction forces acting on the ulnar styloid fragment were not simulated. Therefore, the conclusions of this study are explicitly limited to axial construct behaviour within this simplified experimental model. The physiological transmission of naturally applied forces is not replicated in this model, and particularly the potential contribution of the ulnar collateral ligament tension at the ulnar styloid is not considered. Therefore, clinical outcomes cannot be directly extrapolated from these biomechanical findings. These limitations were accepted in this standardized testing protocol using synthetic bone models to isolate material-related differences without the confounding effects of inter-individual variability inherent to cadaveric studies. The use of synthetic bones also allowed for direct comparison among three fixation groups without requiring paired testing of contralateral specimens. Axial loading parallel to the osteotomy was selected to apply maximal force directly to the tested implants. The differences in group sizes (9 vs. 9 vs. 10) due to exclusion of 2 specimens could have impacted the results. Furthermore, the absence of soft tissue constraints means that the experimental setup may not adequately reflect in vivo biomechanics. The contribution of soft tissue tension to construct stability could not be assessed in this model. It should further be noted that the loading configuration — axial loading parallel to the fracture plane — may inherently favour compression screw fixation, as this direction corresponds to the axis of interfragmentary compression generated by the screw.

Clinical implication.

The fixation method of USF should be considered when determining the postoperative rehabilitation protocol. Based on the superior axial stability demonstrated in this biomechanical model, CHS fixation may theoretically support earlier mobilization; however, clinical rehabilitation protocols should ultimately be guided by individual patient factors, fracture characteristics, and clinical judgment rather than biomechanical data alone. The simplified experimental setup does not allow direct extrapolation to clinical outcomes. Nevertheless, the simplicity, cost-effectiveness, and availability of K-wire fixation justify its continued use in clinical practice. Further studies are necessary to address clinical relevant questions in regards to the technique of USF fixation and the kind of USF that needs osteosynthesis in the first place.

## Conclusion

In summary, among the closed-reduction fixation techniques evaluated in this study, single K-wire fixation provides inferior stability in the osteosynthesis of ulnar styloid fractures. Basal USF with significant instability can be considered for surgical refixation, and based on our biomechanical results, headless compression screw fixation can be deemed as adequate alternative to K-wire fixation when performing closed reduction and internal fixation. Further studies are necessary to evaluate the clinical effects. These findings are limited to axial construct stability within a simplified in vitro model and should not be generalized to the full biomechanical environment of the distal radioulnar joint.

## Data Availability

The datasets used and/or analysed during the current study are available from the corresponding author on reasonable request.

## Abbreviations

1KW: Group with osteosyntheses by one 1.4 mm Kwire
2KW: Group with osteosyntheses by two 1.0 mm Kwires
CHS: Group with osteosyntheses by one cannulated headless screw
DRUJ: Distal radioulnar joint
K-wire: Kirschner wire
mm: Millimetres
N: Newton
SD: Standard deviation
TBW: Tension band wiring
TFCC: Triangular fibrocartilaginous complex
USF: Ulnar styloid fractures
